# Bioanalytical Method Development and Validation of Memantine in Human Plasma by High Performance Liquid Chromatography with Tandem Mass Spectrometry: Application to Bioequivalence Study

**DOI:** 10.1155/2012/101249

**Published:** 2012-03-22

**Authors:** Ravi Kumar Konda, B. R. Challa, Babu Rao Chandu, Kothapalli B. Chandrasekhar

**Affiliations:** ^1^Department of Pharmaceutical Chemistry, Hindu College of Pharmacy, Amaravathi Road, Guntur, Andhrapradesh 522002, India; ^2^Department of Chemistry, Jawaharlal Nehru Technological University, Anantapur 515002, India; ^3^Department of Pharmaceutical Analysis, Nirmala College of Pharmacy, Madras Road, Kadapa, Andhrapradesh 516002, India; ^4^Department of Pharmaceutical Sciences, Donbosco College of Pharmacy, Pulladigunta, Guntur 522201, India

## Abstract

A simple, sensitive, and rapid HPLC-MS/MS method was developed and validated for quantitative estimation of memantine in human plasma. Chromatography was performed on Zorbax SB-C_18_ (4.6 × 75 mm, 3.5 *μ*m) column. Memantine (ME) and internal standard Memantine-d6(MED6) were extracted by using liquid-liquid extraction and analyzed by LC-ESI-MS/MS using multiple-reaction monitoring (MRM) mode. The assay exhibited a linear dynamic range of 50.00–50000.00 pg/ml for ME in human plasma. This method demonstrated an intra- and interday precision within the range of 2.1–3.7 and 1.4–7.8%, respectively. Further intra- and interday accuracy was within the range of 95.6–99.8 and 95.7–99.1% correspondingly. The mean recovery of ME and MED6 was 86.07 ± 6.87 and 80.31 ± 5.70%, respectively. The described method was successfully employed in bioequivalence study of ME in Indian male healthy human volunteers under fasting conditions.

## 1. Introduction

**Figure 1:**
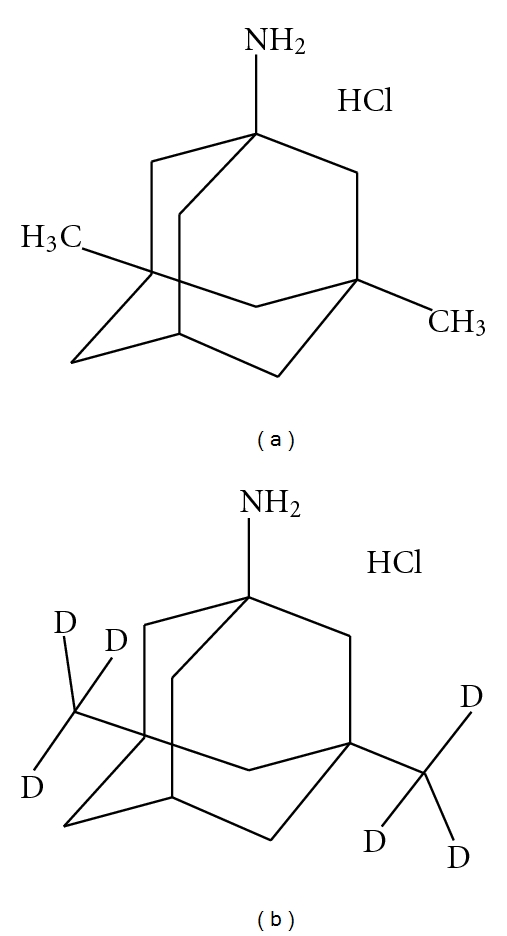
Chemical structures of (a) Memantine hydrochloride and (b) Memantine-D_6_HCL.

Memantine (1-amino-3,5-dimethyladamantane hydrochloride) (Figure 1) acting on the glutamatergic system by blocking N-methyl-D-aspartate (NMDA) glutamate receptors [1]. Memantine (ME) is used in Parkinson's disease and movement disorders, and recently it has been demonstrated to be useful in dementia syndrome. The mode of action is thought to be due to prevention of damage to retinal ganglion as a result of increased intraocular pressure. The accumulation of a drug in melanin-rich tissues may have serious physiological consequences as it could lead to potentially toxic effects. Despite several investigations into the nature of drug melanin binding, the exact mechanism of the interaction remains unknown. ME is well absorbed, with peak plasma concentrations (*C*_max⁡_) ranging from 22 to 46 ng/mL following a single dose of 20 mg. The time to achieve maximum plasma concentration (*T*_max⁡_) following single doses of 10–40 mg ranges from 3 to 8 hr. The drug is 45% bound to plasma proteins presenting a distribution volume of approximately 9–11 L/kg, which suggests an extensive distribution into tissues. It is poorly metabolized by the liver, and 57–82% of the administered dose is excreted unchanged in the urine with a mean terminal half-life of 70 hr [1]. 

There were few methods established previously to determine ME in a variety of matrices with different instruments. These methods include LC-MS [1–4], HPLC [5–8], GC-MS [9], and Micellar electrokinetic chromatography [10]. Among all methods LC-MS [1–4] has gained more importance.

Liu et al. [1] developed the method with the linear concentration range of 0.2–200 ng/mL, with 0.2 ng/mL sensitivity. This sensitivity was improved by Almeida et al. [2]. They developed the method with the linear concentration range of 0.1 to 50 ng/mL, with 0.1 ng/mL sensitivity. Pan et al. [3] developed the method with the linear concentration range of 0.1 to 25 ng/mL. They used 0.5 mL plasma usage to get 0.1 ng/mL of sensitivity. Koeberle et al. [4] developed the method in different melanins.

The reported methods do not show the usage of deuterated internal standard comparision with analyte which is most important in bioanalytical method development. All the reported methods develop the method with long run time and more amount of plasma sample for extraction. 

The purpose of this investigation was to develop a rapid, simple, sensitive, and selective LC-MS/MS method for the quantitative estimation of ME in less volume of human plasma using deuterated internal standard. It is also expected that this method would provide an efficient solution for pharmacokinetic, bioavailability, and/or bioequivalence studies of ME.

## 2. Materials and Methods

### 2.1. Chemicals

ME (99.9%) was obtained from Varda biotech Pvt. Ltd. Andheri, Mumbai, India. MED6 (99.0%) was obtained from the Toronto Research Chemicals, Toronto, Canada. Blank plasma lots were purchased from Navjeevan blood bank, Hyderabad. HPLC-grade methanol and acetonitrile were purchased from Jt. Baker, USA. Diethyl ether and *n*-hexane were purchased from Lab Scan, Asia Co. Ltd, Bangkok, Thailand. Formic acid and sodium hydroxide were purchased from Merck Mumbai, India. HPLC-grade water from Milli-Q System was used. All other chemicals used were analytical grade.

### 2.2. Instrumentation and Chromatographic Conditions

HPLC system (1200 series, Agilent Technologies, Germany) is connected with API 4000 triple quadrupole mass spectrometer (ABI-SCIEX, Toronto, Canada) using multiple reaction monitoring (MRM). A turbo electrospray interface in positive ionization mode was used. Data processing was performed on Analyst 1.4.1 software package (SCIEX). The chromatography was performed on a Zorbax SB-C_18_ (4.6 × 75 mm, 3.5 *μ*m) (Agilent technologies,Germany) at 40°C temperature. The mobile phase composition was a mixture of 0.1% formic acid : acetonitrile (35 : 65 v/v) which was pumped at a flow-rate of 0.6 mL/min without split.

### 2.3. Preparation of Calibration Standards and Quality Control Samples

Standard stock solutions of ME (100.00 *μ*g/mL) and MED6 (100.00 *μ*g/mL) were separately prepared in methanol. MED6 dilution (25.00 ng/mL) was made from MED6 standard stock solution with diluent (methanol: water 50 : 50 v/v). Standard stock solution of ME was added to drug-free human plasma to obtain ME calibration standards of 50.00, 100.00, 500.00, 1000.00, 5000.00, 10000.00, 20000.00, 30000.00, 40000.00, and 50000.00 pg/mL. Quality control (QC) samples were also prepared as a bulk on an independent weighing of standard drug at concentrations of 50.00 (LLOQ), 150.00 (LQC), 15000.00 (MQC), and 35000.00 pg/mL (HQC) from standard stock solutions of ME. The calibration standards and quality control samples were divided into aliquots in 5 mL Ria vials and stored in the freezer at below −30°C until analysis.

### 2.4. Sample Preparation

50 *μ*L of MED6 (25 ng/mL), 100 *μ*L of plasma sample, and 100 *μ*L of 10 mM NaOH were added into 5 mL Ria vials and vortexed briefly. This was followed by addition of 3 mL extraction solvent (diethyl ether : *n*-hexane 70 : 30 v/v) and vortexed for 10 min. Then samples were centrifuged at 4000 rpm for 5 min at ambient temperature conditions. Then, the supernatant from each sample was transferred into labelled vials by using the dry-ice acetone flash freeze technique and evaporated to dryness under nitrogen stream at 40°C. The dried residue was reconstituted with 400 *μ*L of 0.1% of formic acid: acetonitrile (35 : 65 v/v) mixture and vortexed until dissolved. Finally, a 20 *μ*L of each sample was transferred into auto sampler vials and injected into HPLC connected with mass spectrometer.

### 2.5. Recovery

Recovery of ME was evaluated by comparing the mean peak area of six extracted low, medium, and high (150.00, 1500.00, and 35000.00 pg/mL) quality control samples to the mean peak area of six aqueous standards with the same concentrations of low, medium, and high ME quality control samples.

Similarly the recovery of MED6 was evaluated by comparing the mean peak area of extracted quality control samples to the mean peak area of MED6 in aqueous standards samples with the same concentrations of MED6.

### 2.6. Selectivity

The selectivity of the method was determined by blank human plasma samples from six different healthy human volunteers to test the potential interferences of endogenous compounds coeluted with ME and MED6. The Chromatographic peaks of ME and MED6 were identified on the basis of their retention times and MRM responses. The mean peak area of LOQ for ME and MED6 at corresponding retention time in blank samples should not be more than 20 and 5%, respectively.

### 2.7. Limit of Quantification (LOQ)

The LOQ was estimated in accordance with the baseline noise method at a signal-to-noise ratio (S/N) of 5. It was experimentally determined by injecting six samples with ME at the LLOQ concentration. The acceptance criterion for S/N was ≥5 and calculated by selecting the noise region as close as possible to the signal peak, which was at least 8 times of the signal peak width at half height.

### 2.8. Analytical Curves

The analytical curves of ME were constructed in the concentrations ranging from 50.00 to 50000.00 pg/mL in human plasma. The calibration curve was constructed by using instrument response (ratio of ME peak area to MED6 peak area) against the ME concentration (pg/mL) for four consecutive days by weighted 1/*x* ^2^ quadratic regression model. The fitness of calibration curve was confirmed by back-calculating the concentrations of calibration standards.

### 2.9. Calibration Curve Standards, Regression Model, Precision, and Accuracy Batches

Calibration curve standard samples and QC samples were prepared in replicates (*n* = 6) for analysis. Correlation coefficients (*r* ^2^) were obtained by using quadratic regression model in whole range of tested concentrations. The accuracy and precision for the back calculated concentrations of the calibration points should be within ±15% whereas those of LLOQ should be within ±20% of their nominal values.

### 2.10. Stability

Low and high QC samples (*n* = 6) were retrieved from the deep freezer; samples were processed for three freeze/thaw cycles according to the clinical protocols. The samples were stored at −10°C to −30°C in three cycles of 24, 36, and 48 hr. In addition, the long-term stability of ME in QC samples was also evaluated after 76 days of storage at −10 to −30°C. The stability at refrigerated temperature was studied following 79 hr storage period in the autosampler tray. Bench top stability was studied for 26-hour period. Stability samples were processed and extracted along with the freshly spiked calibration curve standards. Stability of the stock solutions was proved for 24 days. The precision and accuracy for the stability samples were maintained within 15 and ±15%, respectively, of their nominal concentrations.

### 2.11. Matrix Effect

The matrix effect due to plasma matrix was used to evaluate ion suppression/enhancement in a signal by comparing the absolute response of QC samples after pretreatment (liquid-liquid extraction) with that of reconstituted samples extracted blank plasma sample spiked with analyte. Experiments were performed at low and high concentration levels in triplicate. The acceptable precision (%CV) should be ≤15%.

### 2.12. Analysis of Human Plasma Samples

The bioanalytical method described previously was applied to determine ME concentrations in plasma following oral administration to healthy adult human male volunteers below 25 years of age. The volunteers were contracted by Micro Therapeutics Research Labs Pvt Ltd., Chennai, India. They were screened before participation in the study and an informed consent was taken from them. These volunteers, were not undergone any other medication before conducting this study. To each of the 20 volunteers a tablet containing 10 mg of ME was orally administered along with a 240 mL of drinking water. Proper diet was provided to each volunteer as per the protocol. The reference product (Namenda tablets 10 mg, Forest laboratories, Ireland) and test product (Memantine tablets 10 mg) were used in the study. The study protocol was approved by IEC (Institutional Ethical Committee) and by ICMR (Indian Council of Medical Research). Blood samples were collected as predose (0 hr) 5 minutes prior to dosing followed by further samples at 1, 2, 3, 4, 4.5, 5, 5.5, 6, 6.5, 7, 7.5, 8, 12, 24, 48, and 72 hr. After dosing, a 5 mL blood sample was collected each preestablished time in vacutainers containing K_2_EDTA. A total of 34 samples (17 time points each for reference and test) were collected and centrifuged at 3200 rpm and10°C for 10 min. Then they were stored at −30°C until further analysis. Test and reference were administered to the same human volunteers under fasting conditions separately after a washing period of 18 days as per protocol approved by IEC.

### 2.13. Pharmacokinetics and Statistical Analysis

Pharmacokinetics parameters were calculated from plasma levels applying a noncompartmental statistics model using WinNon-Lin 5.0 software (Pharsight, USA). Following Food and Drug Administration (F.D.A) guideline [11, 12], blood samples were drawn up to a period of three to five times the terminal elimination half-life (*t*_1/2_) and it was considered as the area under the concentration time curve (AUC) ratio higher than 80%. The *C*_max⁡_ and *T*_max⁡_ values were determined by visual inspection of the plasma ME concentration-time profiles. The area under the concentration-time curve (AUC_0−*t*_) was obtained by the trapezoidal method. The total area under the curve (AUC_0−*∞*_) was calculated up to the last measureable concentration, and extrapolations were obtained by the last measureable concentration and the terminal elimination rate constant (*K*_*e*_). The *K*_*e*_ was estimated from the slope of the terminal exponential phase of the plasma of ME concentration-time curve using linear regression method. The *t*_1/2_ was then calculated as 0.693/*K*_*e*_. The AUC_0−*t*_, AUC_0−*∞*_, and *C*_max⁡_ bioequivalence were assessed by analysis of variance (ANOVA), and the standard 90% confidence intervals (90% CIs) of the ratios test/reference were calculated after transforming the data logarithmically. The bioequivalence was considered when the ratio of averages of log transformed data was within 80–125% for AUC_0−*t*_, AUC_0−*∞*_, and *C*_max⁡_ [11, 12].

## 3. Results and Discussion

### 3.1. Method Development and Validation

**Figure 2:**
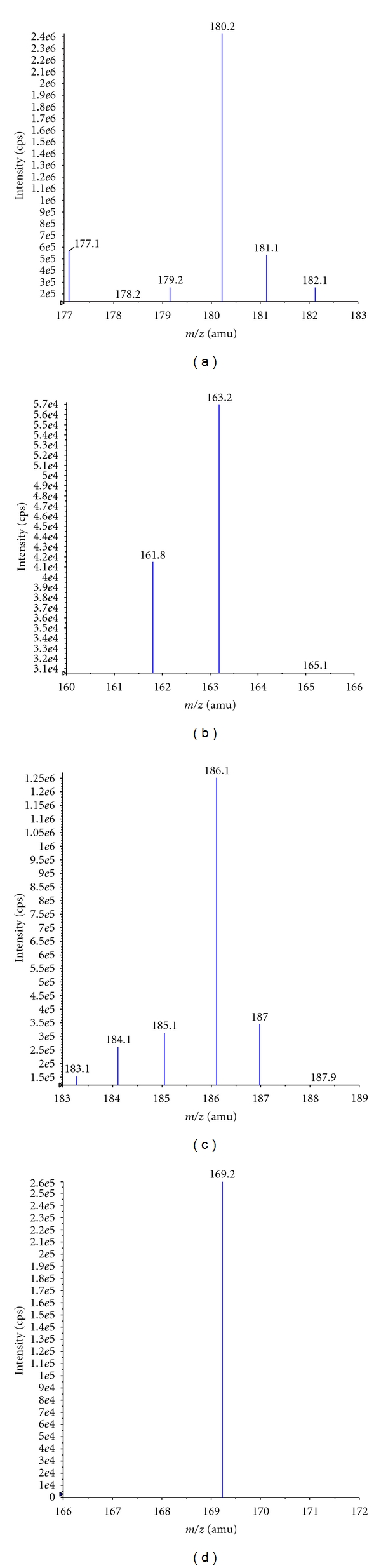
(a) Mass spectra of Memantine parent ion (Q1). (b) Mass spectra of Memantine product ion (Q3). (c) Mass spectra of Memantine-D_6_ parent ion. (d) Mass spectra of Memantine-D_6_ product ion (Q3).

Mass spectrometry parameters, fragmentation pattern, and mode of ionization are the main task in mass spectrometry tuning to obtain respective fragmented ions and response for both ME and MED6 which were shown in Figures 2(a), 2(b), 2(c), and 2(d). ESI-LC-MS/MS is a very powerful technique for pharmacokinetic studies since it provides sensitivity and selectivity requirements for analytical methods. MRM technique was chosen for the assay development. The MRM parameters were optimized to maximize the response for the analyte.

 The instrumental parameters for mass spectroscopy were optimized. The source temperature was 600°C. The gas pressures of nebulizer, heater, curtain, and CAD were 40, 30, 20, and 4 psi, respectively. The ion spray voltage, entrance potential, declustering potential, collision energy, and collision cell exit potential were optimized at 5500, 10, 50, 32, and 12 V, respectively. The dwell time was 400 milliseconds for both ME and MED6.

The product ion (Q3) mass spectra of ME and the MED6 are shown in Figures 2(b) and 2(d). [M + H]^+^ was the predominant ion in the Q1 spectrum. The Q1 for ME and MED6 was 180.2 and 186.1, respectively, and were used as the precursor ion to obtain product ion spectra. The collisionally associated dissociation (CAD) mass spectrum of ME shows formation of characteristic product ions at *m/z *161.8, 163.2, and 165.1. The major product ion at *m/z *163.2 for ME could be explained by the splitting of 1-amino-3-,5-dimethyladamantane hydrochloride from the protonated precursor molecule. The CAD mass spectrum of MED6 shows formation of characteristic product ions at *m/z *169.2. The major product ion at *m/z *169.2 arose from 3,5-Dimethyl-d6-tricyclo-[3,3,1,13,7]decan-1-amine,3,5-Dimethyl-d6-1-adamantanamine from the protonated precursor molecule. The most sensitive mass transitions were from *m/z *180.2 to 1163.2 for ME and *m/z* 186.1 to *m/z* 169.2 for the MED6. The proposed fragmentation pattern is Figure 2(a)→Figure 2(b), Figure 2(c)→Figure 2(d). The inherent selectivity of MS-MS detection was also expected to be beneficial in developing a selective and sensitive method.

 The chromatographic conditions particularly the composition of mobile phase, flow-rate of mobile phase, choosing of suitable column, injection volume, column oven temperature, autosampler temperature, splitting of sample in to ion source, as well as a short run time were optimized through several trials to achieve good resolution and symmetric peak shapes for the ME and MED6. It was found that a mixture of 0.1% formic acid:acetonitrile (35 : 65 v/v) could achieve this purpose and this was finally adopted as the mobile phase. The formic acid was found to be necessary in order to lower the pH to protonate the ME and thus deliver good peak shape. The percentage of formic acid was optimized to maintain this peak shape while being consistent with good ionization and fragmentation in the mass spectrometer. The high proportion of organic solvent eluted both the ME and the MED6 at retention time 1.45 ± 0.2 min at a flow rate of 0.6 mL/min, produced good peak shapes, and permitted a run time of 3.5 min.

Liquid-liquid extraction (LLE) was used for the sample preparation in this work. LLE can be helpful to clean the samples. Clean samples are essential for minimizing ion suppression and matrix effect in LC-MS/MS analyses. Several organic solvents and their mixtures in different combinations and ratios were evaluated. Finally, diethyl ether/*n*-hexane (70 : 30) was found to be optimal, which produced a clean chromatogram for a blank plasma sample and yielded the highest recovery for the ME and MED6 from the plasma. Memantine-D_6_ hydrochloride was used as internal standard for the present purpose. Clean chromatograms were obtained, and no significant direct interferences in the MRM channels at the relevant retention times were observed.

### 3.2. Selectivity

**Figure 3:**
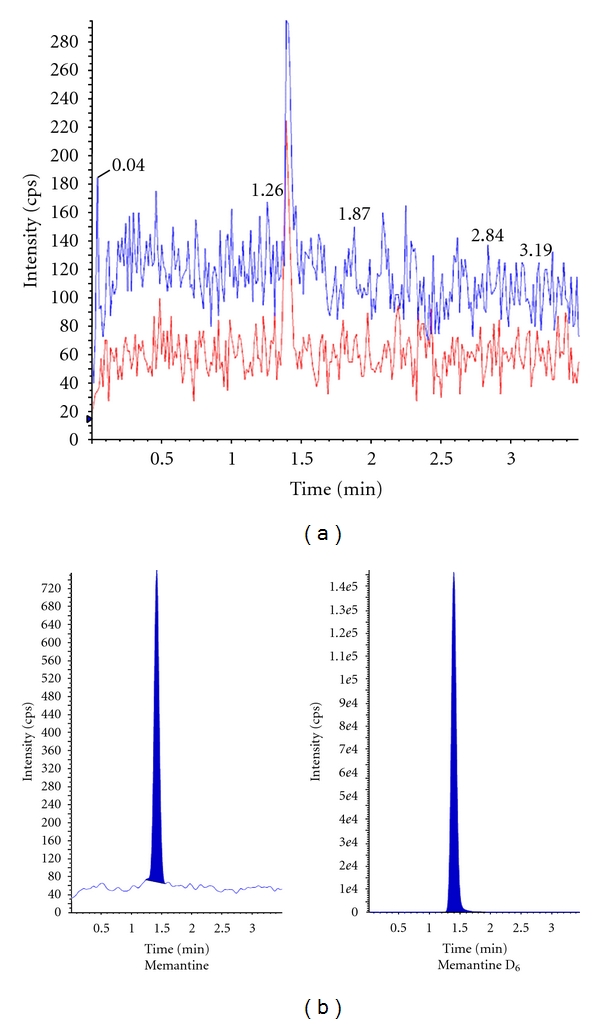
(a) MRM chromatogram for blank plasma. (b) Chromatogram of LOQ.

The selectivity of the method was examined by analyzing blank human plasma extracts (*n* = 6). The result of one blank (Figure 3(a)) plasma is shown and the lack of interference is similar to other samples which were studied which shows no significant direct interference in the blank plasma traces as observed from endogenous substances in drug-free human plasma at the retention time of the analyte.

### 3.3. Limit of Quantification (LOQ)

The LOQ signal-to-noise (S/N) value found for 6 injections of ME at LOQ concentration was 11.93.  Figure 3(b) shows a representative ion-chromatogram for the LOQ (50 pg/mL) with 20 *μ*L injection volume.

### 3.4. Linearity, Precision, and Accuracy

The ten-point calibration curve was linear over the concentration range 50.00–50000.00 pg/mL. The calibration model was selected based on the analysis of the data by quadratic regression with intercepts and weighting factor 1/*x* ^2^. The best quadratic regression for the calibration curve was achieved with a 1/*x* ^2^ weighing factor, giving a mean quadratic regression equation for the calibration curve of *y* = −9.427 × 10^−11^ *x* ^2^ + 9.194 × 10^−5^ *x* + 2.989 × 10^−4^(*y* = *ax* ^2^ + *bx* + *c*) where *y* is the peak-area ratio of the ME to the MED6 and *x* is the concentration of the ME in plasma (Table 1). For the between-batch experiments, the precision and accuracy ranged from 1.4 to 2.7% and 95.7 to 99.1%, respectively (Table 2). Further, in within-batch experiments the precision and accuracy ranged from 2.1 to 2.3% and 95.6 to 99.8% correspondingly.

### 3.5. Recovery

 The recoveries for ME at low (150.00 pg/mL), medium (15000.00 pg/mL) and high (35000.00 pg/mL) plasma concentrations with six replicate injections each showed 79.45 ± 6.20%, 91.25 ± 5.9%, and 87.52 ± 2.59%. The overall recovery of ME was found to be 86.07% ± 6.87%. Similarly extraction recovery of MED6 (25.00 ng/mL) was determined as 80.31% ± 5.70%. Recoveries of ME and MED6 were high, precise, and reproducible. Therefore, the assay has proved to be robust in high-throughput bioanalysis.

### 3.6. Stability Studies

Quantification of the ME in plasma that was subjected to 3 freeze-thaw cycles (−30°C to room temperature) showed the stability of the analyte. The concentrations ranged from 98.00 to 104.00% for ME. No significant degradation was observed even after a 79-hour storage period in the autosampler tray, and the final concentrations of ME were found between 100.00 and 105.00% The room temperature stability of ME in QC samples after 26 hr was also evaluated. The concentrations were ranged between 99.00 and 102.00% for ME. In addition, the long-term stability in low and high QC samples after 76 days of storage at −30°C was also evaluated, and the concentrations ranged from 98.00 to 103.00% for ME. These results confirmed the stability of ME in human plasma for at least 76 days at −30°C. (Table 3).

### 3.7. Application to Biological Samples

**Figure 4:**
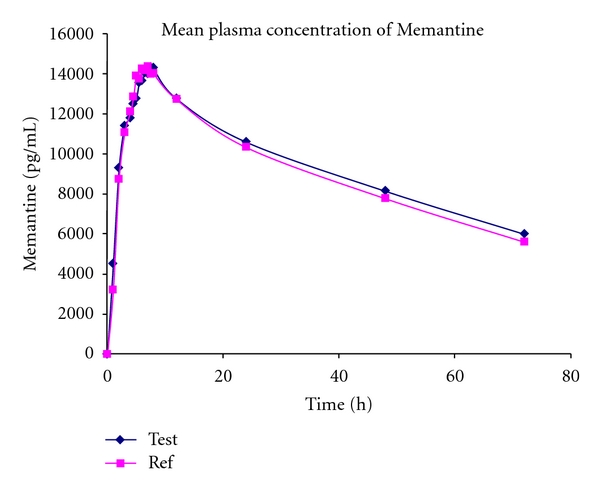
Mean plasma concentrations of test versus reference after a 10 mg dose (one 10 mg tablet) single oral dose (20 healthy volunteers).

The proposed method was applied to the determination of ME in plasma samples for the purpose of establishing the bioequivalence of a single dose (10 mg tablet) in 20 healthy human volunteers. Typical plasma concentrations versus time profiles were shown in Figure 4. Plasma concentrations of ME were in the standard curve range and retained above LLOQ for the entire sampling period. The pharmacokinetic parameters for test and reference products were shown in Tables 4 and 5. The mean ratio of AUC_0−*t*_/AUC_0−*∞*_ was higher than 90% which followed the Food and Drug Administration Bioequivalence Guideline [11, 12]. The ratio of test/reference (T/R) and 90% confidence intervals (90 CIs) for overall analysis were comprised within the previously stipulated range (80–125%). Therefore, it can be concluded that the two ME formulations (reference and test) analyzed are bioequivalent in terms of rate and extent of absorption at fasting conditions.

## 4. Conclusion

A simple, high sensitive, specific, rugged, and reproducible LC-MS/MS method for the determination of memantine in human plasma was developed and validated as per FDA guidelines. This method was successfully applied in bioequivalence study to evaluate the plasma concentrations of ME in healthy human volunteers.

## Figures and Tables

**Table 1:** Concentration data form validation.

Spiked plasma concentration (pg/ml)	Concentration measured (pg/ml) Mean ± Sd *n* = 5	Precision (% CV)	Accuracy %
50.00	49.92 ± 0.44	0.80	99.23
100.00	100.21 ± 1.91	1.90	98.14
500.00	502.73 ± 6.83	1.40	98.65
1000.00	1000.54 ± 11.53	1.10	98.92
5000.00	5005.06 ± 38.75	0.80	99.28
10000.00	9978.95 ± 160.56	1.60	98.45
20000.00	19871.04 ± 303.46	1.50	98.53
30000.00	29759.82 ± 508.47	1.70	98.37
40000.00	40310.88 ± 123.85	0.30	99.74
50000.00	50084.85 ± 266.72	0.50	99.55

**Table 2:** Precision and accuracy (analysis with spiked plasma samples at three different concentrations).

Spiked plasma concentration (pg/ml)	Within-run (*n* = 6)			Between-run (*n* = 30)		
	Concentration measured (*n* = 6) (pg/ml) (mean ± Sd.)	Precision (%CV)	Accuracy %	Concentration measured (*n* = 30) (pg/ml) (mean ± Sd.)	Precision (%CV)	Accuracy %
150.00	143.40 ± 3.20	2.20	95.60	143.50 ± 3.90	2.70	95.70
15000.00	14746.40 ± 338.40	2.30	98.30	14719.10 ± 248.30	1.70	98.10
35000.00	34935.20 ± 730.40	2.10	99.80	34699.20 ± 498.40	1.40	99.10

**Table 3:** Stability of Memantine in human plasma samples.

Spiked plasma concentration (pg/ml *n* = 6)	Concentration measured (pg/ml)	Precision (%CV)	Accuracy (%)
Room temperature stability for 26 hr in plasma			
150.00	151.23	1.00	100.82
35000.00	34926.50	0.80	99.79
Three freeze-thaw cycles			
150.00	153.02	2.30	102.01
35000.00	34818.00	0.90	99.48
Auto sampler stability for 79 hr			
150.00	154.59	1.40	103.06
35000.00	35213.50	0.90	100.61
Stability for 76 days −30°C			
150.00	151.53	3.20	101.02
35000.00	35080.50	1.40	100.23

**Table 4:** Mean pharmacokinetic parameters of Memantine in 20 healthy human volunteers after oral administration of 10 mg test and reference products.

Pharmacokinetic details of Memantine in human plasma		
Pharmacokinetic Parameter	Reference	Test
	Mean ± SD	Mean ± SD
*C*_max⁡_ (pg/ml)	14368.57 ± 4044.16	14328 ± 4324.76
AUC_0−*t*_ (pg·hr/ml)	654545.5 ± 70423.12	674564.4 ± 67858.99
AUC_0−*∞*_ (pg·hr/ml)	1053469.0 ± 77690.79	1136607 ± 74862.04
*T*_max⁡_ (hr)	7.0	7.5
*t*_1/2_	49.29	53.35

AUC_0−*∞*_: Area under the curve extrapolated to infinity.

AUC_0−*t*_: Area under the curve up to the last sampling time.

*C*_max⁡_: The maximum plasma concentration.

*T*_max⁡_: The time to reach peak concentration.

**Table 5:** Pharmacokinetic parameters of memantine after administration of 10 mg of test and reference products in 20 healthy human volunteers.

Pharmacokinetic parameters	*C*_max⁡_ (T/R)	AUC_0−*t*_ (T/R)	AUC_0−*∞*_ (T/R)
Test/Ref	99.72	103.06	107.89
